# Deliberate Play and Preparation Jointly Benefit Motor and Cognitive Development: Mediated and Moderated Effects

**DOI:** 10.3389/fpsyg.2016.00349

**Published:** 2016-03-11

**Authors:** Caterina Pesce, Ilaria Masci, Rosalba Marchetti, Spyridoula Vazou, Arja Sääkslahti, Phillip D. Tomporowski

**Affiliations:** ^1^Department of Movement, Human and Health Sciences, Italian University Sport and Movement “Foro Italico”Rome, Italy; ^2^Department of Kinesiology, Iowa State UniversityAmes, IA, USA; ^3^Department of Sport Sciences, University of JyväskyläJyväskylä, Finland; ^4^Department of Kinesiology, University of GeorgiaAthens, GA, USA

**Keywords:** executive function, variability, enrichment, physical activity, spontaneous play, body weight, children

## Abstract

In light of the interrelation between motor and cognitive development and the predictive value of the former for the latter, the secular decline observed in motor coordination ability as early as preschool urges identification of interventions that may jointly impact motor and cognitive efficiency. The aim of this study was twofold. It (1) explored the outcomes of enriched physical education (PE), centered on deliberate play and cognitively challenging variability of practice, on motor coordination and cognitive processing; (2) examined whether motor coordination outcomes mediate intervention effects on children’s cognition, while controlling for moderation by lifestyle factors as outdoor play habits and weight status. Four hundred and sixty children aged 5–10 years participated in a 6-month group randomized intervention in PE, with or without playful coordinative and cognitive enrichment. The weight status and spontaneous outdoor play habits of children (parental report of outdoor play) were evaluated at baseline. Before and after the intervention, motor developmental level (Movement Assessment Battery for Children) was evaluated in all children, who were then assessed either with a test of working memory (Random Number Generation task), or with a test of attention (from the Cognitive Assessment System). Children assigned to the ‘enriched’ intervention showed more pronounced improvements in all motor coordination assessments (manual dexterity, ball skills, static/dynamic balance). The beneficial effect on ball skills was amplified by the level of spontaneous outdoor play and weight status. Among indices of executive function and attention, only that of inhibition showed a differential effect of intervention type. Moderated mediation showed that the better outcome of the enriched PE on ball skills mediated the better inhibition outcome, but only when the enrichment intervention was paralleled by a medium-to-high level of outdoor play. Results suggest that specifically tailored physical activity (PA) games provide a unique form of enrichment that impacts children’s cognitive development through motor coordination improvement, particularly object control skills, which are linked to children’s PA habits later in life. Outdoor play appears to offer the natural ground for the stimulation by designed PA games to take root in children’s mind.

## Introduction

There is a widespread belief that being physically active is intrinsic to children’s behavior. Nevertheless, tracking studies show that trends of inactivity have an early onset at pediatric age (Telama, 2009), with children not meeting the recommended physical activity (PA) guidelines already at preschool age (Tucker, 2008). Secular trends of PA decline are accompanied by a noteworthy decline in physical fitness (Tomkinson and Olds, 2007; Runhaar et al., 2010) and, as early as preschool age, also in motor fitness measured in terms of motor coordination and fundamental motor skills (Roth et al., 2010; Vandorpe et al., 2011). While apparently less relevant for health, motor skill competence is considered critical for meeting PA recommendations later in life (Barnett et al., 2008; Stodden et al., 2008), since persons who have not mastered fundamental motor skills in childhood are more likely to refrain from participating in organized sports and PA. Therefore, motor coordination is emerging as a relevant public health factor (Iivonen and Sääkslahti, 2014; Robinson et al., 2015).

The relationship between PA and motor skill competence has been proposed to be reciprocal. That is, children with higher levels of perceived and actual competence are more likely to engage in PAs, which, in turn, may lead to further skill development and increased perceived and actual competence (Robinson et al., 2015). On the other hand, children with motor difficulties are less inclined to participate in PA, thus limiting their opportunities for practice and further development (Skinner and Piek, 2001). This virtuous or vicious circle has an impact not only on motor development, but also on social, emotional, and cognitive development.

Indeed, longitudinal non-interventional evidence has shown that the development of fundamental motor skills at preschool age predicts social and emotional development (Bart et al., 2007; Piek et al., 2008a), cognitive efficiency and academic achievement (Piek et al., 2008b; Niederer et al., 2011; Roebers et al., 2014) in the subsequent phase of transition to school. This intriguing line of research (van der Fels et al., 2015 for a review) provides evidence on the linkage of motor coordination and skill competence to cognitive efficiency and academic achievement in children (Lopes et al., 2013; Haapala et al., 2014) and adolescents (Rigoli et al., 2012a,b; Marchetti et al., 2015). This interrelation between motor and cognitive developmental trajectories at behavioral level is consistent with neuroscientific evidence on the close parallelism and interaction of the neural substrates of motor coordination and cognitive executive function (Diamond, 2000; Koziol et al., 2011). However, to establish causality, needed is interventional research that assesses how motor and cognitive developmental outcomes are jointly influenced by PA. To this end, particular interest has been devoted to the effects of exercise on children’s executive cognitive functions (Tomporowski et al., 2011) that are responsible for goal-oriented behaviors, cognitive flexibility and behavioral adaptability (Etnier and Chang, 2009).

In extensive reviews of interventions aiding executive functions, Diamond and Lee (2011) and Diamond and Ling (2015) stated that structured PA programs focusing on both motor skill development and cognitive engagement have a stronger impact on children’s cognitive function than computerized programs without physical effort or physical (aerobic or resistance) training without a cognitive component. The authors also highlighted that to obtain improvements executive functions must not just be used, but continually challenged. Consistent to this, the link between coordinated actions and cognition and the brain substrate that acts as an interface between these domains emerges particularly evident in the study of complex and therefore challenging motor coordination tasks (Serrien et al., 2007).

Coordinative and cognitive complexity of movement tasks have been proposed as potential mechanisms through which PA impacts executive function efficiency beyond the more commonly studied role of exercise-related metabolic and physiological changes (Best, 2010). The idea of cognitive stimulation by coordinatively and cognitively engaging movement tasks has been further developed in terms of ‘gross-motor cognitive training’ (Pesce, 2012), designed PA and sports characterized by novelty and diversification (Moreau and Conway, 2014) and tailored to aid cognition and metacognition (Tomporowski et al., 2015a), sensorimotor training or physically active mindfulness practices that require ‘thoughtful moving’ (Ben-Soussan et al., 2015b; Diamond, 2015).

Interventional exercise and cognition studies performed with preadolescent children have a large variety of qualitative PA characteristics (Vazou et al., in press). In some studies, the effect of the coordinative and cognitive challenges of the movement tasks was not disentangled from that of the aerobic exercise intensity, either because the study focused on exercise quantity and dose-response relations (Davis et al., 2011; Hillman et al., 2014), or because coordinative/cognitive engagement was deliberately combined with aerobic exercise or with enhanced PA time to reap largest cognitive benefits (Chang et al., 2013; Reed et al., 2013; Crova et al., 2014; van der Niet et al., 2016). In other studies, the comparison was between aerobic exercise, which is usually performed with movement tasks requiring low coordinative and cognitive engagement (e.g., running), and traditional PE, which is usually centered on motor skill development and learning through lower-intensity exercise tasks (e.g., object control skills, Fisher et al., 2011).

The notion that adding coordinative/cognitive complexity to PA or adding PA to cognitive tasks is beneficial to cognitive performance is supported by few recent studies. They employed more than two intervention arms or held exercise intensity constant between cognitively more or less challenging PA conditions to disentangle spurious effects (Pesce et al., 2013b; Mavilidi et al., 2015; Schmidt et al., 2015). However, none of them has searched for mechanisms that can explain if there is a causal linkage between motor and cognitive outcomes of ‘enriched’ movement tasks. The present study was aimed at addressing this issue. Cognitive executive functions and motor skills are fundamentally interrelated at both the levels of behavior and brain substrate (Koziol et al., 2011) and this relationship especially emerges when complex motor coordination comes into play (Serrien et al., 2007). Thus, we hypothesized that an enriched PA intervention with coordinative and cognitive challenges embedded in playful activities would have positive outcomes on executive function linked to motor coordination outcomes.

A second aim of the present study was to evaluate whether eventual benefits of the enriched PA intervention are affected by lifestyle factors such as spontaneous outdoor play habits and weight status. The studies on enriched PA effects on children’s cognition have been mainly performed in physical education (PE). However, PA experiences in a structured context as PE are only one side of the coin. Play that occurs in natural outdoor environments stimulates variation in children’s movements and at the same time effectively acts as physical training (Fjortoft, 2004). An actual issue of debate is whether the dichotomy of spontaneous play vs. deliberate practice may be reconciled either proposing a common framework (Côté and Hancock, 2016), or identifying intermediate forms of play and practice that bridge the two extremes (MacNamara et al., 2015). We hypothesize that structuring PE in form of deliberate play and deliberate preparation, that emphasize enjoyment and participation in a variety of play activities (Côté et al., 2007) and focus on fundamental motor skills acquisition by means of developmentally appropriate tasks (Giblin et al., 2014), respectively, may promote a reciprocal influence and transfer effects between PE and spontaneous play. According to transfer taxonomy (Barnett and Ceci, 2002), while the physical and functional contexts are different (school vs. playground), the modality of learning and learned procedural skills are similar (hands-on discovery and problem-solving heuristic). Thus, it was hypothesized that spontaneous play might amplify the effectiveness of our PE program centered on deliberate play and preparation. This effect was hypothesized to occur not only in the motor skills domain, but also in the cognitive domain, since executive function may benefit from the enjoyable and physical nature of the activities (Pellegrini and Smith, 1998; Diamond and Ling, 2015).

As regards weight status, evidence shows both motor and cognitive disadvantages of overweight children as compared to their lean peers (Krombholz, 2013; Reinert et al., 2013). Longitudinal non-interventional research shows that in the absence of targeted intervention, the evolution of motor coordination over time is closely related to children’s weight status, with an increasingly wider gap between overweight and lean children’s coordination along development (D’Hondt et al., 2013). Thus, we evaluated whether the enriched PA environment may represent a means to support the development of children who are at risk of poor coordination and cognition.

## Materials and Methods

The research program, developed as a part of a Corporate Social Responsibility commitment for PA promotion for children, was approved by the Ethics Committee of the “Umberto I” hospital of the First Rome University and authorized by the provincial and regional PE School Offices, the Committees of the school involved and children’s parents, who gave written informed consent. Bilateral agreements were signed between the regional School Office, the University and the corporate.

### Participants

**FIGURE 1:**
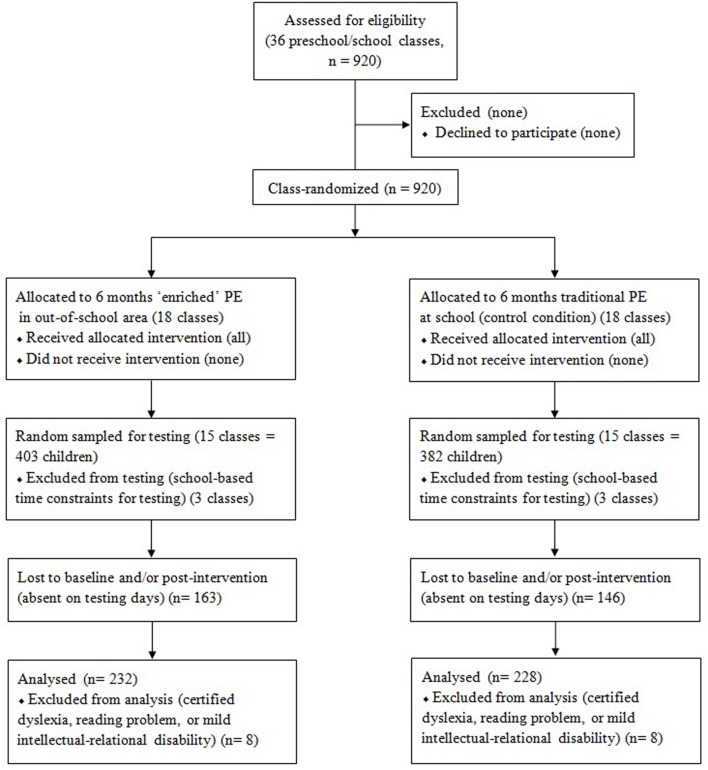
**Study flow diagram for the group-randomized trial**.

Nine hundred and twenty preschool and primary school children aged 5–10 years (480 boys and 440 girls) belonging to eight schools of the municipality of Alba (Italy) volunteered. In the ecological school setting schools and classes, but not individuals could be recruited. Thus, the present study design was group (cluster) randomized, with children in the same school and class sharing the same environment. The progress through the phases of enrollment, intervention allocation, follow-up, and data analysis is represented in **Figure 1**.

As local community stakeholders (e.g., provincial School Office, corporate) supported the research project, the rate of declining by school principals, personnel and parents was 0%. Within schools, 36 classes were stratified random sampled, with six classes for each school grade (last preschool year and primary school grades) to be randomly assigned to the enriched or traditional PE (three classes each). The final sample was composed of 460 children, aged 5–6 (163), 7–8 (144), and 9–10 years (153), with 232 (115 males and 117 females) belonging to the classes assigned to the enriched PE and 228 (115 males and 113 females) belonging to the traditional PE classes.

Reduction of participants from those assessed as eligible was due to school logistics (e.g., adapting testing time constraints; 17% random sampled and excluded) and to a data loss (e.g., children’s absence at one or more baseline and/or post-intervention testing days; 39%). Since those children were balanced across intervention and control classes, no intent-to-treat analysis was performed. As concerns missing data, we evaluated (1) the number of cases missing per variable and (2) the number of variables missing per case. (1) No individual variables were missing data for more than half the cases and percentage missing cases were balanced among variables (M-ABC variables: 20% at baseline plus 3% at post-test; weight status and outdoor play at weekend: 21% each). (2) 15% of the cases were missing data for more than half of the variables. The likelihood of children being absent on testing days was probably not influenced by differences in basic demographic characteristics or socio-economic status, which, though not assessed because considered sensitive information, are presumed limited in a small-size town. Furthermore to limit data loss at post-test to children who changed school/residence or were absent from school for prolonged periods, two possible testing days were planned for each test 1 week apart.

### Intervention Characteristics

All children assigned to the intervention or control group participated in PE for 1 h once a week. The teacher–child ratio was about 1:25 in the control classes, as prescribed by Italian legislation, but was altered in the experimental classes due to the additional presence of the external specialist teacher. The intervention lasted 6 months, from November to April, preceded and followed by testing in October and May, respectively. Neither teachers, nor children could be blinded after assignment to the intervention or control group. In fact, only the classroom generalist teachers of the intervention classes took actively part to teacher training together with PE specialist teachers to realize the intervention program cooperatively during the curricular school time. Children were aware to be part of the intervention group, because the intervention was jointly led by an external PE specialist teacher and the classroom teacher in an out-of-school area rendered available by the municipality and equipped with sports facilities and managed by the corporate.

Both the generalists teaching in the control classes and those teaching in the intervention classes together with the PE specialists taught PE according to the age-related PE goals defined in the Italian curriculum for kindergarten and primary school, mainly focused on motor and social skills development (Ministry of Education, Universities and Research (MIUR), 2013). To ensure ecological validity of the control condition, the generalists teaching in the control classes did not receive any particular instruction by the researchers. Instead, those teaching in the experimental classes underwent teacher training together with the specialists once a month for 4 h to prepare the forthcoming teaching module, analyze teaching behaviors of the previous module and address interdisciplinary issues. To this aim, in all classes participating to the intervention, one lesson was videotaped for use as teacher training material.

There were four teaching modules, each lasting 6 weeks, with a total amount of 24 intervention hours. From a holistic view on education though movement based on the assumption that PE is critical to educating the whole child (National Association for Sport and Physical Education, 2011), all modules jointly pursued following improvement goals: physical fitness, motor coordination, cognition, life skills (Pesce et al., 2015). The modules were differentiated in that each of them addressed specific aspects of fitness, coordination, cognition and life skills through age-appropriate PA games. As an example in the first module, the focus was on the cardiovascular component of physical fitness, the perceptual-motor adaptation component of coordinative ability, the cognitive flexibility component of executive cognitive function and the goal setting component of life skills. Typical PA games that may jointly challenge these components are tag games. While any type of tag games involves moderate-to-vigorous running intensities that challenge the cardiovascular system and changes in running direction and speed according to changes in the environment that challenge perceptual-motor adaptation, the employed tag games were altered to enhance the demands on cognitive flexibility and goal setting. To stimulate cognitive flexibility, fixed roles for taggers and non-taggers where substituted by dual roles: in a ‘rock, paper and scissors game’-like fashion, children (e.g., rock-children) had to tag some mates (e.g., scissors-children), while avoiding to be tagged by other mates (e.g., paper-children) (Tomporowski et al., 2015b).

To generate mental engagement and specifically stimulate executive function and motor coordination, PA games emphasized variability of practice (Pesce et al., in press) and contextual interference as applied in motor skill learning to deliberately keep children on the learning curve (Tomporowski et al., 2010). To ensure that the PA games were specifically tailored to challenge core executive functions, they were created based on instructional principles of executive tasks used in cognitive developmental research (Huizinga et al., 2006; Garon et al., 2008). According to the claim that interventions aiding executive functions work best when increasingly demanding and emotionally loaded (Diamond and Lee, 2011), we employed games characterized by age-appropriate variations in task complexity and emotionally engaging social interactions. Also, open-ended tasks were employed in which only the starting point, the rule(s) and the task goal were indicated and children were encouraged to find many possible solutions to perform it. This type of games challenges motor creativity that seems to rely on inhibitory efficiency (Scibinetti et al., 2011).

In sum, the PA games employed in this intervention had characteristics of deliberate play (Côté et al., 2007) and deliberate preparation (MacNamara et al., 2015). Cognitive teaching strategies and enjoyable problem solving conditions targeted to promote exploration and participation (typical for deliberate play) were developed and constrained in ways, exemplified above, that ensured the systematic stimulation of different facets of motor and life skill competence (typical for deliberate preparation). Cognitively challenging task demands were not only an issue of PE content, but also of delivery. In fact, they were generated employing a constraints-led approach to motor problem solving in which children alternately searched for ‘the’ optimal solution, or for a wider range of pertinent solutions, alternating repetition and change, stabilization and destabilization of movement patterns (Renshaw et al., 2010; Pesce et al., in press).

In the attempt to ‘quantify’ the qualitative characteristics of teaching and estimate their fit with the approach described above, stratified random sampling was performed to select four intervention and four control classes balanced as to preschool/school levels that were videotaped during a PE session for analysis of teaching behaviors. The qualitative intervention features were categorized as behavioral categories of teaching strategies (Rink, 2006) (**Table 1**) by two independent raters, experienced in video-analysis and categorization of teaching behaviors. The outcomes of the categorization, quantified as percentage (%) of events for time unit (20 s) during PE, were submitted to reliability computation. A satisfactory intra- and inter-observer agreement, indicated by a percentage of agreement [Agreements/(Agreements + Disagreements) × 100] ≥ 0.80, was reached.

**Table 1:** Category definitions for teaching strategies (Rink, 2006).

Teaching strategies	
Interactive teaching	Teaching strategy most commonly used in PE. The instructional process is controlled by teacher who is responsible for the selection and progression of the content, for task communication (usually to an entire group), feedback and evaluation.
Peer teaching	Instructional strategy according to which some teacher’s responsibilities are transferred to the student. The teacher usually maintains responsibility for content selection and progression, but uses one student to show or ‘teach’ a skill to another and to provide reciprocal feedback and evaluation.
Cognitive strategies	Group of teaching strategies designed to engage the learner cognitively in the content by producing solutions rather than reproducing any movement pattern they have been shown by teacher. This is an umbrella term for problem solving, guided and divergent discovery, and teaching through questions.
Cooperative learning	Teaching strategy according to which the goal to be achieved is meaningful and the task requires team work to be fulfilled, with process and product of the cooperative learning experience being evident to the learners.

Since the aim of the present study was to evaluate whether ‘enriching’ PE quality without enhancing its quantity would lead to joint coordinative and cognitive benefits, we needed to control for potential covariation of PA type and intensity. Thus, physical exercise intensity was indirectly monitored during 20 PE lessons of 10 experimental and 10 control, stratified for school grades, classes by recording heart rate (HR) with HR monitors (Polar S610i). According to the available amount of HR monitors, data were recorded on a total subsample of 127 children, similarly distributed among school grades and between intervention and control classes (*n* = 67 vs. 60, respectively). Mean HR and percentage of time spent in moderate or vigorous PA were calculated on the available subsample of HR data. The time spent in moderate or vigorous PA was operationalized as 140 ≥ HR ≥ 159 and HR ≥ 160 bpm, respectively (Wang et al., 2005).

### Instruments and Procedures

Prior to the intervention, children’s body height and weight and PA level were measured objectively and their outdoor play was estimated by means of a questionnaire completed by parents. Following familiarization to tests protocols, participants were assessed by trained experimenters at baseline and after the 6 months intervention period. Pre-post intervention assessments included a test of motor developmental level (Movement Assessment Battery for Children) administered to all children and tests of cognitive efficiency administered to two subsamples. Half of the children performed a test of working memory (Random Number Generation task, RNG) and half performed a test of attention (belonging to the Cognitive Assessment System, CAS). Cognitive testing was not preceded by PA lessons to avoid acute exercise effects. The same procedures and schedule were applied for pre- and post-intervention testing, maintaining the same order and time for test administration (always in the morning).

#### Weight Status and Spontaneous Outdoor Play Assessment

Children’s body height and mass were measured for BMI (kg/m2) calculation. Age-referenced cut-off values of BMI were used to identify overweight and lean children (Cole et al., 2000). The percentage of children classified as overweight was similar in the sample of children initially random sampled for testing (25,6%) and in the final sample used for analysis (27,4%).

Children’s spontaneous play habits in outdoors environments were estimated by means of the Children’s Outdoor Play assessment questionnaire (Veitch et al., 2009). It consists of eight items for weekdays and eight for weekend. Parents were requested to report the number of days their child spent playing in locations as yard at home, friend’s/neighbor’s yard, own street/court/footpath, park/playground in out-of-school hours on weekdays and weekend days during a typical week. Weekday responses were based on a five-point scale ranging from never/rarely to 5 days per week, and weekend day responses were on a six-point scale ranging from never/rarely to every Saturday and Sunday. Parents were asked to count only the days where their child spent at least 10 min in a specific location, considering 10 min an appropriate minimum time span. The validation of the Italian version is described in the Preliminary analyses section.

Recently, it has been recommended to use parental-reports of PA only as context-specific measures, since they seem not useful as a proxy for children’s general PA levels (Verbestel et al., 2015). Since for all children of all schools enrolled, a playground was rendered accessible on Sundays by the corporate for playing outdoor with their parents, to properly draw conclusions limitedly to the context-specific play behavior, only scores of outdoor play at weekend were used for main analyses.

#### Motor Coordination Assessment

To assess children’s motor coordination performance, the Italian version of the Movement Assessment Battery for Children (M-ABC) developed by Henderson and Sudgen (1992) was used. The more recent version of the (M-ABC-2, Schulz et al., 2011) could not be used because at the pre-test time (October 2013) the M-ABC-2 still was not available in the Italian version. This test evaluates movement competence providing objective quantitative data on both gross motor and fine motor coordination of children aged from 4 to 12 years with eight tasks differentiated in four age-related difficulty levels. The M-ABC has been proved to be a valid and reliable research and diagnostic tool that covers the entire domain of motor ability and is used to identify motor problems and DCD (Croce et al., 2001). The tasks are grouped under three subheadings: manual dexterity (three tasks), ball skills (aiming and catching, two tasks), and static and dynamic balance (one and two tasks, respectively).

##### Manual dexterity

The first task is ‘posting coins’ (5–6 year-old), ‘placing pegs’ (7–8 year-old), or ‘shifting pegs by rows’ (9–10 year-old). The child must drop coins through the slot in a bank box, or place 12 plastic pegs in all holes on a board, or move pegs from a given row to another, respectively, one at a time as quickly as possible. The second task is ‘threading beads’ (5–6 year-old), ‘threading lace’ (7–8 year-old), or ‘threading nuts on bolt’ (9–10 year-old). The child must thread beads through a lace, or thread a lace back and forth through the holes in a lacing board, or screw nuts down a bolt, respectively, one at a time as quickly as possible. In both the first and second tasks, the examiner measures the seconds taken to complete each task. The third task is ‘bicycle trail’ (5–6 year-old), or ‘flower trail’ (7–10 year-old). The child must draw with the preferred hand one continuous line following the bicycle or flower trail on a record form without crossing its boundaries. The examiner records the number of errors, i.e., the number of times the drawn lines moves outside a boundary.

##### Ball (aiming and catching) skills

The first task is ‘catching bean bag’ (5–6 year-old), ‘one-hand bounce and catch’ (7–8 year-old), or ‘two-hand catch’ (9–10 year-old). The child must catch a bean bag tossed by the experimenter, or bounce a tennis ball on the floor and catch it with the same hand, or throw a tennis ball at the wall form behind a marked line and catch it at the return with both hands, respectively. The second task is ‘rolling ball into goal’ (5–6 year-old), or ‘throwing bean bag into box’ (7–10 year-old). The child must roll a tennis ball along the floor between two stands to score a ‘goal,’ or throw a bean bag into a target box on the floor form behind a marked line. In both types of tasks, the examiner records the number of correctly executed trials (successful catches and throws) out of 10 attempts.

##### Static and dynamic balance

The task evaluating static balance is ‘one-leg balance’ (5–6 year-old), ‘stork balance’ (7–8 year-old), or ‘one-board balance’ (9–10 year-old). The child must stand on one leg, with the arms held at the sides, or stand on a foot, place the sole of the other foot against the side of the supporting knee and the hands on the hips, or balance on one foot on a balance board, respectively. The examiner records the number of seconds up to 20 the child maintains balance. The second task is ‘jumping over cord’ (5–6 year-old), ‘jumping in squares’ (7–8 year-old), or ‘hopping in squares’ (9–10 year-old). The child must jump over the cord from a stationary position, or make five continuous jumps forward from a starting square to further five squares, or make five continuous hops forward from square to square on one foot, respectively. The examiner records if the child performs a successful jump (‘jumping over cord’) or the number of correct consecutive jumps/hops completed over five without performance errors (‘jumping or hopping in squares’). The third task is ‘walking heels raised’ (5–6 year-old), ‘heel-to-toe walking’ (7–8 year-old), or ‘ball balance’ (9–10 year-old). The child must walk along a line with heels raised without stepping off the line, or placing the heel of one foot against the toe of the other, or walk around the outside of two stands and return to the starting point while steadying a board with a ball in the middle of it, respectively. The examiner records the number of steps performed by the child without leaving space between toe and heel or stepping off the line.

##### Data coding and scoring

For each of the three subheadings of manual dexterity, ball (aiming and catching) skills, and static and dynamic balance, the data were transformed into scores of impairment of motor function according to age-related normative data (Henderson and Sudgen, 1992). Each impairment score indicates the extent to which a child falls below the level of his/her age peers, while it does not differentiate between children who perform above this level. Test–retest reliability data were not collected, as high reliability results are available for children across all age groups considered in this study (Henderson and Sudgen, 1992; Croce et al., 2001).

#### Assessment of Executive Cognitive Function and Attention

##### Inhibition and working memory updating

To assess two core executive functions (inhibition and working memory updating), children individually performed the RNG task (Towse and Neil, 1998), lasting about 10 min, which has been proven feasible also with children 5 year-old and over (Towse and McIachlan, 1999). They were told that the RNG is a game involving numbers and were instructed to verbally generate a random sequence of numbers between 1 and 10 to each beat of a 70-beat sequence with an inter-beat interval of 1.5 s. Prior to data collection by tape recording, participants performed a familiarization trial of 70 numbers and could ask questions concerning the test. Both the omission of a number generation in correspondence of one tone and the production of numbers lower than 1 (0) or higher than 10 (11, 12 etc.) were considered errors and discarded. If errors exceeded a predefined maximum threshold of five, the entire block was repeated.

The randomness of the sequence of numbers was measured by means of 18 different indices described by Towse and Neil (1998). Among those, five indices were selected as they reflect two components of executive function (Miyake et al., 2000): the ability to inhibit mental routines (turning point index [TPI], adjacency score [Adj], and runs score [Runs]) and the ability to update and manipulate information held in working memory (redundancy score [Red], and mean repetition gap [MeanRG]). A third index of working memory (coupon score [Coupon]), updating was excluded because its association to the other two indices (Red and MeanRG) was not confirmed in children (Crova et al., 2014).

The computation and meaning of these indices is extensively described in Audiffren et al. (2009). In short, the TPI is a ratio between the real frequency of turning points between ascending and descending series of numbers (e.g., the response change between the digits “2” and “5” in a hypothetical sequence “9, 7, 2, 5, 6, 8”) generated by the participant and their theoretical frequency in random responses. The Adj measures the relative frequency of pairs of adjacent ascending or descending numbers (e.g., 7–8 or 4–3) as compared to the total number of response pairs produced by the participant. The Runs score is an index of variability of the number of digits in successive ascending or descending runs. The Red index reflects the unbalance of response alternative frequencies in a sequence that derives from a more frequent usage of given numbers than expected based on the theoretical frequency of each digit in random responses. The MeanRG is the mean number of responses given until each digit reoccurs calculated for all digits throughout the whole sequence (e.g., in the sequence “2, 8, 4, 6, 2, 9, 7, 8,” the digits “2” and “8” reoccur with a mean gap equal to 4).

Turning point index, Adj and Runs were merged into an average index of inhibition, while Red and MeanRG into an average index of memory updating. High levels of TPI, but low values of Adj and Runs reflect a good ability to suppress the habitual tendency to count forward or backward, as well as high levels of MeanRG, but low levels of Red reflect a good ability to update information on already generated or still not generated digits held in working memory. Thus before averaging, all indices were z standardized and Adj, Runs, and Red were reversed (Crova et al., 2014).

##### Attention

To assess attention, the tasks belonging to a subscale of the CAS were used (Naglieri and Das, 1997, Italian version 2005). The CAS consists of 12 subtests that assess four aspects of cognition: Planning, Attention, Simultaneous and Successive processes (PASS theory) (Das et al., 1994). For the present study, because of school time constraints, participants only performed the Attention tasks, which is relevant for learning and sensitive to cognitively challenging PA games (Pesce et al., 2013a). We did not collect test-retest reliability data, since acceptable to good reliability data are available for children of the age considered in this study (Naglieri and Das, 1997). The Attention scale is composed of three subtests that require the child to use focal attention to detect target stimuli and avoid distractions. The raw score for each subtest is converted to an age-based standard score and summed to obtain a total scale value.

The first subtest, Expressive Attention, is a Stroop-like test composed of three items that measures attention selectivity and interference control under time pressure. The first and the second items are without interference condition, while the third is with interference. There are two age-specific sets of items. For example, in the version for children 8 years and older, the non-interference conditions are reading color words (Blue, Yellow, Green, and Red) all written in dark ink and naming the colors of a series of rectangles (printed in blue, yellow, green, and red). In the interference condition, the words Blue, Yellow, Green, and Red are printed in a different color ink than the colors the words name and the child is instructed to name the color ink the word is printed in, rather than to read the word. Only this last item is used as the measure of attention.

The second subtest, Number Detection, measures selectiveness and capacity to resist distraction under time pressure. It is comprised of pages of numbers where the child must underline the correct numbers among a large quantity of distracters in different formats. For example, the child must find a particular stimulus (the numbers 1, 2, and 3 printed in an open font) on a page containing many distracters (the same numbers printed in a different font style). The raw score is the ratio of the accuracy (total number correct minus the number of false detections) and the time to completion summed across the items.

The third subtest, Receptive Attention, is a two-page subtest that measures the ability to focus and then shift attention between different stimulus dimensions under time pressure. On the first page, children must identify and underline pairs of target letters that are physically identical (e.g., TT but not Tt), whereas on the second, pairs of letters that have the same name (e.g., Aa not Ba) are targets to be underlined. For all subtests, the raw score is the ratio of the accuracy (total number correct in the first subtest and total number correct minus the number of false detections in the second and third subtests) and time to completion summed across items/pages.

## Preliminary Analyses

Prior to main analyses, (1) the Italian version of the children’s Outdoor Play assessment questionnaire was validated; (2) the quantitative and qualitative characteristics of PE lessons, as well as the enjoyment perceived by children in the intervention and control classes were contrasted to control for potential covariates of the qualitative characteristics of PE lessons. Specifically, it was controlled for potentially higher exercise intensity due to the expertise of PE specialist teachers and higher enjoyment due to the presence of the specialist teacher and the novelty of the out-of-school area and facilities that might explain eventual differential motor and cognitive outcomes in the intervention and control classes.

### Validation of the Outdoor Play Assessment Questionnaire

The children’s outdoor play assessment questionnaire (Veitch et al., 2009) was translated and back-translated to ensure adequacy of translation. Prior to the administration to the parents of the children who participated in the present study, the questionnaire was administered to a further sample of 71 parents twice, one week apart to verify its test–retest reliability, internal consistency, and criterion-based validity. Test–retest reliability was acceptable (Cronbach’s α = 0.79) only after removing the eighth item (play in an unspecified location). Internal consistency, as assessed by ICC computation, was at least moderate (ICC = 0.58–0.79) for all items. The criterion-based validation was performed on the base of the information delivered by a 7-day diary, where parents were asked to indicate on each of the nominated 7 days whether their child had played in specified outdoor locations (the same as in the likert-scale questionnaire) in after-school hours for at least 10 min. Kappa statistic (κ) and percent agreement between responses were used to assess validity. Kappa values for each specified outdoor location were moderate (range: 0.408–0.574).

### Quantitative and Qualitative Analysis of Physical Education Lessons

The type of teaching strategies and the intensity of physical exercise during the recorded PE lessons are reported on **Table 2** for comparison between intervention and control classes. The main difference was that in generalist-led traditional PE lessons, interactive teaching was prioritized, whereas in the lessons led by specialists in cooperation with generalist classroom teachers, cognitive teaching strategies as problem solving and teaching through questions prevailed, representing more than half of the total teaching time. In general, in the intervention classes, there was a more differentiated use of teaching strategies, while in the control classes the use of teaching strategies different from the interactive was negligible.

**Table 2:** Teaching styles (mean % of events for 20-s time unit), physical exercise intensity (mean heart rate and percentage of time spent in moderate or vigorous physical activity) and perceived enjoyment during representative PE lesson types: traditional, generalist-led and enriched, specialist-led.

	Traditional generalist-led (Control)	Enriched specialist-led (Intervention)
**Teaching strategies**	**% Events**	**% Events**

Interactive	87.7	25.3
Peer teaching	5.7	11.3
Cognitive	5.2	54.2
Cooperative	1.4	9.2

**Exercise intensity**		

Mean HR (bpm ± SD)	132.2 (±23.5)	131.9 (±17.4)
PE time spent in moderate PA (% ± SD)	22.5 (±12.1)	25.9 (±12.4)
PE time spent in vigorous PA (% ± SD)	16.7 (±15.5)	22.2 (±17.4)

As regards exercise intensity, pairwise comparisons (*t*-tests) for independent samples did not yield significant differences between the intervention and the control group in mean HR, *t*(125) = 0.18, *p* = 0.855 and % time spent in moderate PA, *t*(125) = 1.56, *p* = 0.120, or in vigorous PA, *t*(125) = 1.88, *p* = 0.062 (**Table 2**).

## Results

### Analysis of Intervention Effects

The statistical analyses that follow were performed with IBM SPSS statistics 23. We (i) tested for baseline differences in all outcome and moderator variables that might influence the intervention outcomes; (ii) analyzed the effects of the traditional vs. enriched PE intervention. Descriptive statistics are reported in **Table 3**.

**Table 3:** Baseline and post-intervention level (mean ± SD) of motor coordination (for the total sample, *n* = 460), executive function and attention variables (two halves of the sample, *n* = 230, respectively) of 5–10 year-old children assigned to traditional or enriched PE lessons, led by the generalist teacher (G-led) or PE specialist teacher (S-led) in cooperation with the generalist.

		Traditional PE (G-led)	Enriched PE (S-led)
**Manual dexterity**			
(impairment score)	*pre*	5.9 ± 3.3	6.0 ± 3.5
(improvement ↓)	*post*	5.0 ± 3.6 ˆ***	3.7 ± 3.1
**Ball (aiming/catching) skills**			
(impairment score)	*pre*	3.2 ± 2.9	3.0 ± 2.7
(improvement ↓)	*post*	1.7 ± 2.2ˆ***	1.0 ± 2.8
**Static/dynamic balance**			
(impairment score)	*pre*	4.2 ± 3.9ˆ**	5.3 ± 4.0
(improvement ↓)	*post*	2.6 ± 3.2ˆ***	1.7 ± 2.7
**Inhibition**			
(std. total score)	*pre*	-0.08 ± 2.5	-0.34 ± 2.3
(improvement ↑)	*post*	-0.60 ± 2.3ˆ*	1.11 ± 1.6
**Working memory updating**			
(std. total score)	*pre*	-0.07 ± 1.8	0.29 ± 1.8
(improvement ↑)	*post*	-0.06 ± 0.7	0.10 ± 0.6
**Attention**			
(scale sum score)	*pre*	25.1 ± 7.6	23.6 ± 7.6
(improvement ↑)	*post*	33.0 ± 8.0	30.0 ± 8.3
**Expressive attention**			
(subscale score)	*pre*	8.9 ± 3.2	8.3 ± 3.3
(improvement ↑)	*post*	11.3 ± 3.7	10.2 ± 3.5
**Number detection**			
(subscale score)	*pre*	7.7 ± 3.1	7.4 ± 3.0
(improvement ↑)	*post*	10.4 ± 2.9	10.2 ± 2.8
**Receptive attention**			
(subscale score)	*pre*	8.6 ± 3.2	7.9 ± 3.2
(improvement ↑)	*post*	11.2 ± 3.3	10.6 ± 3.5

*^∗^p < 0.05; ^∗∗^p < 0.01; ^∗∗∗^p < 0.001.*

(i) Pre-intervention impairment scores for manual dexterity, ball (aiming and catching) skills and static/dynamic balance (measured in the whole sample, *n* = 460), as well as average indices of inhibition and working memory updating and sum score for attention (measured in either of two sample halves) were submitted to multivariate analysis of variance (MANOVA) and subsequent ANOVAs. PE intervention (traditional vs. enriched) was the between-subjects factor. The MANOVA performed on motor coordination data showed a significant difference between groups at pre-test [Wilks *λ* = 0.97, *F*(5,454) = 2.68, *p* = 0.021, *$\eta_{p}^{2}$* = 0.03]. ANOVAs revealed that this difference was significant only for static/dynamic balance [*F*(1,458) = 9.05, *p* = 0.003, *$\eta_{p}^{2}$* = 0.02], with lower impairment score for the traditional PE group. The MANOVA performed on executive function indices (inhibition, working memory updating) and on individual attention subscales and sum score did not yield any significant difference between traditional and enriched PE types (*p_s_* > 0.40). Also, neither tested moderators showed significant baseline differences between intervention and control classes (BMI: 17.2 ± 2.7 vs. 16.9 ± 2.6; outdoor play at weekend: 19.2 ± 6.3 vs. 19.6 ± 6.4, respectively).
(ii) After checking for potential baseline differences, we run the analysis of intervention effects. A general linear model was applied to post-intervention measures of the same motor and cognitive development variables, adding their counterpart baseline measures and age as covariates, with PE intervention (traditional vs. enriched) as main factor and gender, weight status (lean vs. overweight) and spontaneous outdoor play (low vs. high) as potential moderators of intervention effects. The nested model that is usually adopted for cluster-randomized data was deemed unsuitable for the present data set. Schools could not be nested within PE intervention groups because both intervention and control classes were stratified-random sampled in each participating school. Nesting children within classes would render the study underpowered because the average number of children tested in each of the 30 classes was relatively low (15) and above all unbalanced among classes.

**FIGURE 2:**
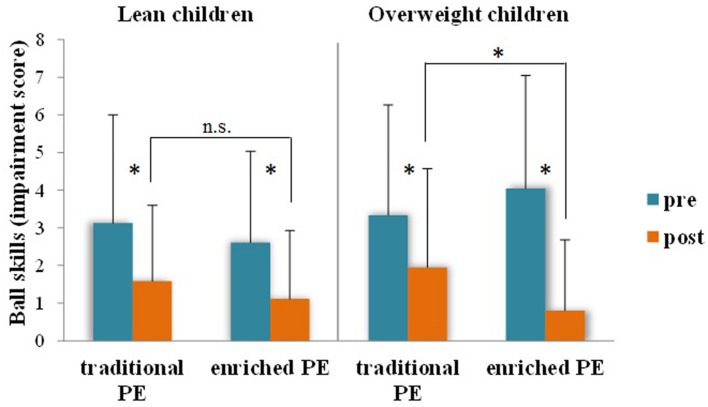
**Ball skills (impairment score – the lower, the better, ±SD) before and after traditional or enriched PE types as a function of children’s weight status**. ^∗^*p* < 0.008; n.s., non-significant.

**FIGURE 3:**
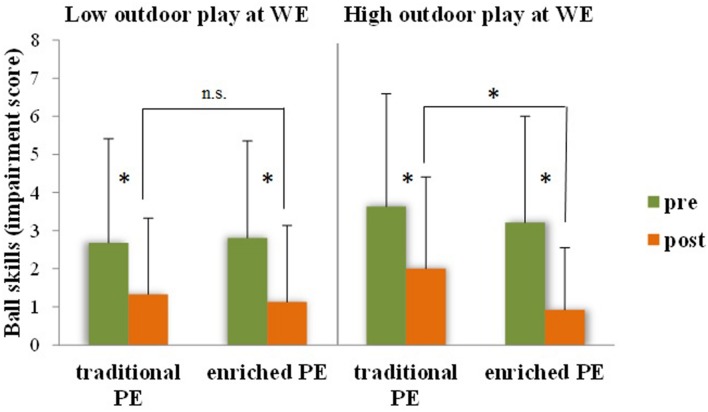
**Ball skills (impairment score – the lower, the better, ±SD) before and after traditional or enriched PE types as a function of children’s spontaneous outdoor play habits at weekend.** ^∗^*p* < 0.008; n.s., non-significant.

Children assigned to the ‘enriched’ intervention showed more pronounced improvements (i.e., larger reduction of the impairment score) in all motor coordination assessments (**Table 3**): manual dexterity [*F*(1,442) = 13.28, *p* < 0.001, *$\eta_{p}^{2}$* = 0.03], ball skills [*F*(1,442) = 23.57, *p* < 0.001, *$\eta_{p}^{2}$* = 0.05], static/dynamic balance [*F*(1,442) = 15.41, *p* < 0.001, *$\eta_{p}^{2}$* = 0.03]. Moreover, as regards ball skills, there were significant interactions of intervention type with weight status [*F*(1,442) = 11.65, *p* = 0.001, *$\eta_{p}^{2}$* = 0.03] and outdoor play at weekend [*F*(1,442) = 8.07, *p* = 0.005, *$\eta_{p}^{2}$* = 0.02]. *Post hoc* analysis of the interactions (*t*-tests, adjusted *p* for six comparisons 0.008) showed that the effect of intervention type on ball skills was independently moderated by weight status and outdoor play in an amplifying manner. Pre-post differences were more pronounced and the advantage of the enriched PE group in ball skills at post-test was significant when children were overweight (**Figure 2**, *p* = 0.006) and, regardless of weight status, when they were involved in outdoor play at a medium-to-high level during weekend days (**Figure 3**, *p* < 0.001). Among executive function and attention variables, only inhibition showed a differential effect of intervention type [*F*(1,220) = 5.55, *p* = 0.019, *$\eta_{p}^{2}$* = 0.03], with higher post-intervention values for the enriched intervention than the traditional PE type. No main effect for gender emerged and gender didn’t either moderate any of the differential effects of enriched and traditional PE. Analogous intervention effects on the same motor coordination and inhibition variables were obtained applying the change score method that is using pre-post delta scores.

### Analysis of Mediating Mechanisms

The statistical analyses that follow were performed with PROCESS macro for SPSS. Cognitive performance (i.e., inhibition) was differentially affected by PE intervention type. Thus, it was entered into multiple mediation analysis (Hayes, 2013) to evaluate whether and to what extent the motor coordination outcomes of PE enrichment may be responsible for cognitive performance outcomes. In the case of ball skills, which were affected by the enriched PE intervention type in a moderated manner, a moderated mediation model was also applied.

#### Multiple Mediation

To test for mediation, regression analyses were performed on post-intervention data to assess the effects of:

(1) the independent variable (X: PE intervention type) on the dependent variable (Y: inhibition);
(2) the independent variable (X: PE intervention type) on each mediator (M: manual dexterity, ball skills, static/dynamic balance);
(3) the independent variable (X) and the potential mediators (Ms) on the dependent variable (Y).

The potential mediators were entered simultaneously to include the covariances among them and the independent variable in the regression equation. The parallel multiple mediator model allowed estimating if introducing all potential motor developmental mediators reduced the direct effect of the PE intervention on the efficiency of the inhibitory function (i.e., total indirect effect of X on Y through Ms).

Then, bootstrapping was applied to empirically estimate the sampling distribution of the indirect effect and generate a bootstrap confidence interval (95% CI) based on 10,000 bootstrap samples for bias corrected bootstrap CIs. This CI was used as a form of hypothesis test to estimate if the size of the indirect effect of each individual mediator, as well as the difference between the indirect effects of the mediators were different from zero (Hayes, 2013).

**FIGURE 4:**
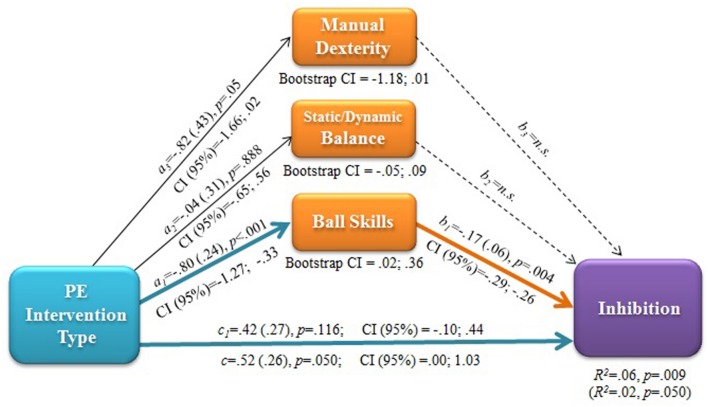
**Multiple mediation model: effects of PE intervention type on post-intervention inhibitory efficiency mediated by post-intervention level of ball (aiming and catching) skills.** a, b, c: regression coefficients with (SE), p and CI (95%) values. c: total effect; a_1_^∗^b_1_, a_2_^∗^b_2_, a_3_^∗^b_3_: indirect effects; c1: direct effect after accounting for mediators. R^2^ values with/without mediators (in parentheses) and bootstrap CI (95%) for indirect effects are also reported.

Significant results of the multiple mediation analysis are reported in **Figure 4**. The post-intervention improvement in ball skills, but not that in manual dexterity or static/dynamic balance, was found to mediate the beneficial effect of the enriched PE intervention on inhibitory function. This is indicated by the bootstrapping output: Only in the case of the ball skills variable, the 95% CI of bootstrap estimates of the indirect effect did not include the zero value (lower bound 0.02, upper bound 0.36). The bootstrap CI for the pairwise comparisons between the indirect effects of the three tested mediators yielded estimates of effect size differences statistically different from zero for the contrast Manual dexterity minus Ball skills (lower bound -0.43, upper bound -0.03), confirming the larger effect of Ball skills. The same mediation model was applied to pre-intervention data and to pre–post delta scores (absolute values and delta magnitude expressed in standard deviation units), but did not yield any significant result.

#### Moderated Mediation

Ball skills were the only significant movement-related mediator of cognitive outcomes of the intervention. In addition, the effect of the intervention on ball skills was moderated by weight status and outdoor play. Thus, a moderated mediation with a conditional indirect effect was also tested (Hayes, 2013). Specifically, it was estimated whether the mediating role of ball skills on the relationship between intervention type and inhibition outcomes was moderated by the fact that children were lean/overweight, or more/less used to playing outdoor at weekend.

**FIGURE 5:**
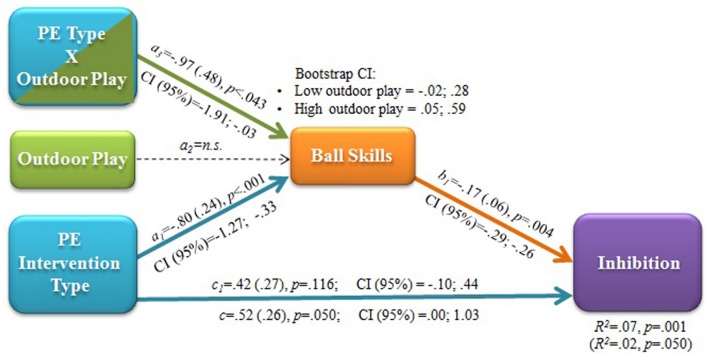
**Statistical model of moderated mediation: effects of PE intervention type on post-intervention inhibitory efficiency mediated by post-intervention level of ball (aiming and catching) skills.** a, b, c: regression coefficients with (SE), p and CI (95%) values. c: total effect; c_1_: direct effect after accounting for the conditional indirect effect of ball skills moderated by outdoor play. R^2^ values with/without mediators (in parentheses) and bootstrap CI (95%) for indirect effects at low vs. high levels of the moderator are also reported.

Spontaneous outdoor play, but not weight status, resulted to be a significant moderator of the mediated path (**Figure 5**). The post-intervention outcome in ball skills found for the enriched PE intervention mediated the inhibition outcome. However, this mediation occurred only when the enriched intervention was paralleled by a relatively high level of outdoor play. This is indicated by the bootstrap 95% CI of the indirect effect not including the zero value only for high outdoor play and by the outcome of the test of equality of the conditional indirect effect in the two groups (lower bound -02, upper bound 0.54).

## Discussion

The present study aimed to further our understanding of the complex relationship between motor and cognitive development. It addressed the question of whether PA experiences centered on variability of practice (Pesce et al., in press) that join coordinative and cognitive challenges in a playful way may act as environmental enrichment that benefits children’s higher-level cognition, the executive, and whether cognitive benefits are mediated by the motor coordination outcomes of the enrichment. It was also examined whether the lifestyle factors of outdoor play habits and weight status moderate the effects of the enriched PA intervention. In sum, the findings showed that enrichment in PE elicited small-size, but wide-ranging improvements of gross motor and fine motor skills and selective improvement of a core executive function—inhibitory processing (**Table 3**). The improvement of object control (aiming and catching) skills was also sensitive to lifestyle factors. The beneficial effect of participating to the enriched PE intervention compared to traditional PE was more pronounced for children, who also were habitual outdoor players (**Figure 3**). Spontaneous outdoor play habits appear to offer the natural ground for the stimulation by deliberate play to take root in children’s mind, since the joint effect of outdoor and deliberate play in PE on object control skills resulted to mediate the effect on inhibitory efficiency (**Figure 5**).

### Enriched Physical Activity as a Unique Means to Promote Motor and Cognitive Development

In several countries (as Italy in the present study), legislation for preschool and primary school levels is not supportive of sufficient amounts of PE that remains far below the minimum threshold to increase children’s fitness level (Strong et al., 2005). That is why the target of school PE in such countries should be creating prerequisites for practice of and lifelong adherence to PA in out-of-school settings and for development and learning in motor and cognitive domains. The renewed attention to the link between motor and cognitive development has been paralleled by a shift, in exercise and cognition research, from the quantitative view on dose-response relations to a qualitative view on coordinative and cognitive movement task demands that may impact cognition (Best, 2010; Pesce, 2012). This has prompted researchers to create PA intervention programs in PE that stimulate children’s executive functions and provide evidence on the beneficial ‘side effects’ of thoughtful PA experiences on the developing brain (Diamond, 2015; Diamond and Ling, 2015; Tomporowski et al., 2015a; Vazou et al., in press). Our study supports the suitability of age-appropriate, thoughtful PA games that exploit principles of variability of practice and deliberate play and preparation to promote motor and cognitive development jointly (Tomporowski et al., 2015b; Pesce et al., in press).

The broad range of intervention effects on different facets of motor coordination skills (manual dexterity, ball skills, balance) supports the view that variability of practice may act as a form of enrichment that broadly affects motor coordination (Pesce et al., 2013b). This outcome may also depend on the fact that our games joined characteristics of deliberate play, emphasizing exploration, playfulness and fun (Côté et al., 2007), and features of deliberate preparation, supporting and facilitating the acquisition of fundamental motor and non-motor skills needed for proactive participation and learning success (MacNamara et al., 2015). In other words, the play experiences were well- and not ill-defined, as variability of practice and problem solving were constrained in ways that directed exploration to match the different objectives planned for the sequential teaching modules. Intriguing commonalities with the outcomes of other large-scale studies employing a well-defined intervention program regard the improvement of motor skills (Piek et al., 2013) and the reduction of hyperactive and inattentive behaviors that rely on inhibitory efficiency (Piek et al., 2015). Piek et al.’s (2010) “Animal fun” program, centered on deliberate play, incorporated variability of practice and task complexity and was structured in sequential modules specifically targeting all gross-motor and fine-motor skills in a playful social interaction context. Playfulness that is reported to be a positive determinant of children’s motor skills (Iivonen and Sääkslahti, 2014) is naturally linked to enjoyment, which is considered relevant also for reaping cognitive benefits from PA (Diamond and Ling, 2015; Pesce and Ben-Soussan, 2016), as demonstrated with children in an ecological learning context (Vazou and Smiley-Oyen, 2014).

We found the enriched PE program beneficial for the inhibitory component of cognitive executive function, but not for working memory updating or attention, suggesting differential associations between specific aspects of motor competence and specific executive processes. Previous research had shown that children’s ability to overcome interference or suppress mental routines may benefit from PE experiences that have inherent cognitive and social interaction demands (Crova et al., 2014; van der Niet et al., 2016). In those studies, however, PE had been enhanced in both quantity and quality. The present study isolated the effects of the qualitative component without altering the low dose of PE prescribed by the country legislation. One of the few studies tailored to disentangle spurious effects of aerobic exercise and cognitive challenges by movement found different effects compared to the present study and particularly no effects on inhibitory processes (Schmidt et al., 2015). This inconsistency, probably due to the team sport-based characteristics of Schmidt et al.’s intervention, suggests that the type of cognitive outcomes may depend on the type of coordinative and cognitive demands of the PA tasks. From a developmental perspective, this inconsistency may be related to the fact that different executive functions ‘come online’ at different time points along child development, with inhibition being the first to be fully developed and therefore more prone to changes in the early stages of cognitive development (Best and Miller, 2010).

The absence of differential intervention effects on attention as measured with the attention scale of the CAS is in line with the outcomes of some studies in which the quantity of PA had been manipulated (Davis et al., 2011; Fisher et al., 2011), but not other studies. Pesce et al. (2013a), for example, found that cognitively challenging PE benefited children’s attention and that the benefits on different aspects of attention depended on children’s motor developmental status. Future research is needed to ascertain the role of individual characteristics as moderators of the exercise-attention relation. The absence of enhanced PA effects on children’s attention assessed with the CAS is also at odds with neuroscientific evidence of the beneficial effects of PA and fitness on task performances requiring executive attention (Pesce and Ben-Soussan, 2016). The lack of convergent findings calls for further research conducted with a broader set of executive and non-executive attention measures to distinguish between spurious and truly executive attention outcomes. The absence of effects on children’s ability to manipulate information in working memory suggests that the central executive component of working memory may not be sensitive to cognitive stimulation by movement in preadolescent children (Crova et al., 2014). Rather, it seems related to the fitness outcomes of larger amounts of PA (Marchetti et al., 2015), as consistently found for hippocampal-dependent associative memory (Khan and Hillman, 2014).

### Cognitive Outcomes of Enriched Physical Activity as a ‘Side Effect’ of Motor Competence

The novel and most interesting result is the unique causal relation linking the intervention outcomes on object control skills and inhibitory function (**Figure 4**). To our knowledge, no study has evaluated whether cognitive benefits obtained with cognitively enriched PA games are merely paralleled, or mediated by positive outcomes in motor coordination. We found evidence of mediation, supporting the hypothesis that beyond the direct cognitive stimulation via physical movement, there may be an indirect causal pathway that links the exposure to enriched PA to motor coordination outcomes that turn into more efficient executive function.

Although the enriched PE caused significantly larger gains in motor coordination and inhibition efficiency than the traditional PE, the extent to which inhibition improved was not explained by gains in motor coordination, as indicated by the absence of a mediation path between delta scores of motor skills and inhibitory function. In the ecological school context, several factors may have influenced children’s baseline inhibitory performance and its changes over time, impeding to see a significant relationship between intervention-related gains in inhibitory efficiency and motor skills. However, a mediating mechanism was found to link motor (object control) skill and inhibitory performance after the intervention, but not before it. Thus, whatever the size of the motor skill and executive function gains, the enriched PE seems to “align” specific aspects of cognitive performance to specific motor skill competence.

The linkage between motor skills and inhibitory control has been also evidenced by cross-sectional studies (Haapala et al., 2014). The present finding adds a causal direction to this linkage and highlights the specificity in the type of motor skills involved. This specificity is also supported by cross-sectional evidence, suggesting some association of object control skills with executive inhibitory function, but not with attention (van der Fels et al., 2015). However, the interpretation of this relationship is tentative as inhibition is multifaceted in nature; different aspects of response inhibition (inhibition of prepotent responses, of mental routines, and interference control) might differently relate to motor control (Livesey et al., 2006). For instance, Castelli et al. (2006) found an association between the interference control aspect of inhibitory function and object control skills as assessed with sport-like ball skills. Our mediational results suggest the existence of shared mechanisms underpinning such relationship. Although consistent evidence of association between object control skills and working memory is reported (van der Fels et al., 2015), our enriched PA intervention failed to impact the central executive of working memory as assessed by the RNG task.

Based on our findings, we contend that the development of object control skill competence is not only a primary underlying mechanism that promotes long-lasting engagement in PA (Barnett et al., 2009; Robinson et al., 2015 for a review), but also a mechanism that partially explains why enriched PA benefits executive function. We hypothesize that executive inhibitory control is involved in and therefore challenged by the intentional control and environmental interaction needed to perform aiming and catching tasks. Promoting the development of ball skills has been shown beneficial to executive processes as planning, that relies on inhibitory efficiency (Westendorp et al., 2014), thus confirming the relevance of visuomotor adaptation experiences for recruiting prefrontal regions that support inhibition (Gentili et al., 2013). Trends in cognitive neurosciences that view cognition as subserving action and being grounded in sensorimotor interaction (Engel et al., 2013; Ben-Soussan et al., 2015a) are inspiring novel research on motor learning in ecological PA and sport settings as a means for cognitive training (Moreau and Conway, 2014) also for children in the school setting (Venditti et al., 2015). Our deliberate play and preparation approach centered on variability of practice (Pesce et al., in press) belongs to this emerging line of research.

It is also to consider that there were a large amount of teacher training and a consequently different use of teaching strategies by teachers of intervention and control classes (**Table 2**). Thus, the cognitive challenges that we assume responsible for the intervention outcomes were the resultant of interrelated content (coordinatively and cognitively engaging PA games) and delivery (cognitive teaching strategies as problem solving, guided and divergent discovery). Our PA games emphasized the roundtrip between repetition and change, between stability and flexibility, providing problem-solving opportunities and boundaries of exploration, manipulating key constraints on learners according to a constraints-led approach to motor learning that fits with the principles of deliberate play and preparation (Renshaw et al., 2010; Pesce et al., in press). In sum, a key feature of our multicomponent, holistic approach to motor and cognitive development promotion centered on deliberate play is the inextricability of cognitive task complexity and teaching strategies to generate complexity in a playful way.

### The Converging Role of Environmental Enrichment and Lifestyle Factors

A further novelty of the study is the finding that educational intervention and lifestyle factors interactively contribute to determine object control competence and that this interaction also explains the mediated effect of PA enrichment on inhibitory efficiency. An intriguing study showed that a structured PA intervention at school was more beneficial for enhancing cognition than free play activities (Fredericks et al., 2006). However, the authors examined the effects of spontaneous play and structured PA practice independently from each other in two intervention arms. Instead, we tailored the intervention to promote converging effects of spontaneous (outdoor) and deliberate (intervention) play practice and found that spontaneous play at outdoor locations seems to amplify the benefits of the enriched PE intervention on ball skills (**Figure 3**). In fact, we designed PA games centered on deliberate play, which is activity done for its own sake, and deliberate practice, which is child-appropriate wide-ranging promotion of fundamental skills development. Both are characterized by flexibility and enjoyment and targeted to bridge spontaneous and deliberate practice (Côté et al., 2007; MacNamara et al., 2015). In this way, we attempted to generate continuity between spontaneous play and deliberate play activities in the enriched PE context. We did so based on the assumption that if strongly structured PA tasks were employed in the intervention, we would reduce both the feasibility of such experiences to be transferred to a spontaneous play context and the cognitive engagement needed to deal with open–ended, discovery learning tasks. The outcome of the moderated mediation model (**Figure 5**) suggests that the value added by spontaneous play extends from the motor to the cognitive domain.

The fact that the intervention effect on object control skills was largest for overweight children highlights how an enriched PA environment may provide equal development opportunities to children who are at risk of developing motor and cognitive disadvantage (D’Hondt et al., 2013; Krombholz, 2013; Reinert et al., 2013). In contrast to what expected based on previous evidence (Crova et al., 2014), the observed benefit of PA enrichment for overweight children emerged in the motor, but not in the cognitive domain and did not moderate the mediational path linking the PE intervention to its cognitive outcome through motor competence outcome. However, given the predictive value of object control competence for PA levels later in life (Robinson et al., 2015), playing enriched PA games may represent a means to promote positive developmental trajectories of health also for overweight children. This is in line with the increasing attention, at academic, as well as at policy level, for the value of play as a health factor (Alexander et al., 2014), contributing to the prevention of Exercise Deficit Disorder (“Play now or pay later,” Faigenbaum and Myer, 2012, p. 196).

### Limitations

The study has limitations to be addressed. Neither children, nor teachers could be blinded. Therefore, the intervention outcomes might be biased by a Hawthorne effect that is the tendency to a higher engagement if being involved in an experimental intervention. Particularly, a higher engagement by children must be taken into account because the deliberate play intervention emphasized variability of practice and was performed in an out-of-school area equipped with sports facilities. The felt novelty and diversity probably caused a higher enjoyment and motivation (Sylvester et al., 2014) that may have contributed to the motor and cognitive benefits of the enriched PE intervention. However, it is difficult and beyond the scope of this holistic approach to children’s motor and cognitive development promotion to disentangle enjoyment effects from those of variability used as a means of embodied cognitive training (Pesce et al., in press), since bodily motion, emotion and cognition are strictly intertwined (Diamond and Ling, 2015; Pesce and Ben-Soussan, 2016).

A further limitation is that the amount of explained variance both in the analysis of intervention effects and in the mediation model was small and the direct effect of PA intervention on inhibition remained significant after accounting for the meaningful indirect effect by the ball skills gain. This suggests that enriched PA has only a small, though significant positive impact on cognitive functioning and multiple factors beyond motor coordination may mediate this benefit. However, the high reach and adoption and the broad range of outcomes counterbalances the low effect size of significant outcomes. Finally, the parental report of outdoor play is not an optimal indicator of children’s physically active play activity (Verbestel et al., 2015). It is possible that the lack of measures that encompass a wide range of children’s movements has made it difficult to show the convergent relation between motor skills and PA on cognitive development (Laukkanen et al., 2014). Finally, the large amount of missing data and the restrictive strategy used may have limited the statistical power of the study. However, given the large sample size and the absence of drop-out from participation, the reason of missing at testing days is primarily attributable to the need to find an adequate trade-off setpoint between research and school organizational needs.

### Outlook and Future Directions

The unique characteristic of the present interventional study was its multicomponent nature. It incorporated various elements from different types of training programs into PA games. Those elements, such as cognitive and social stimulation embedded into enjoyable activities, have been proven suitable to aid executive function in children (Diamond and Lee, 2011). This enriched PA interventions implemented in natural school environment showed how cognitively challenging games during school PE lessons may support executive functions without decreasing PA intensity. The present intervention is inspired by the idea to employ deliberate play and preparation activities that can bridge spontaneous play and deliberate practice along the continuum of PA modalities (Côté et al., 2007; MacNamara et al., 2015). Moreover, it follows the call to ground synergistic commitments for PA promotion for children on the provision of their right to play (Leone et al., 2015) that has been repeatedly acknowledged by authoritative institutions (United Nations, 2013). Considering the emerging public health position on the role of “Playing for health” (Alexander et al., 2014, p. 155), future directions of applied research at the intersection of prevention science, developmental psychology, and cognitive neuroscience (Bryck and Fisher, 2012) should add quality PA into the equation.

## Author Contributions

CP: Main role in the conception and design of the work, data analysis and interpretation, drafting of the work, final approval of the version to be published and agreement to be accountable for all aspects of the work. IM: Data acquisition and analysis with relevant role in data acquisition coordination, drafting of the work, final approval of the version to be published and agreement to be accountable for all aspects of the work. RM: conception and design of the work with relevant role in project coordination, data interpretation, critical revision of the work for important intellectual content, final approval of the version to be published and agreement to be accountable for all aspects of the work. SV: Data interpretation, drafting and critical revision of the work for important intellectual content with specific contribution as regards the mechanisms of cognitive stimulation by designed PA, final approval of the version to be published and agreement to be accountable for all aspects of the work. AS: Data interpretation, drafting of the work with specific contribution as regards motor developmental contents, final approval of the version to be published and agreement to be accountable for all aspects of the work. PDT: data interpretation, critical revision of the work for important intellectual content with specific contribution as regards the chronic exercise-cognition relationship in children, final approval of the version to be published and agreement to be accountable for all aspects of the work.

## Conflict of Interest Statement

The authors declare that the research was conducted in the absence of any commercial or financial relationships that could be construed as a potential conflict of interest. Financial support from the Ferrero Group within the Kinder+Sport pillar of its Corporate Social Responsibility initiatives was received for the research project the submitted work belongs to. However, the funders had no role in the collection, analysis and interpretation of data; in the writing of the report; and in the decision to submit the article for publication.
